# The effects of repetition frequency on the illusory truth effect

**DOI:** 10.1186/s41235-021-00301-5

**Published:** 2021-05-13

**Authors:** Aumyo Hassan, Sarah J. Barber

**Affiliations:** 1 Department of Psychology, San Francisco State University, 1600 Holloway Avenue, Ethnic Studies & Psychology Building, San Francisco, 94132 California USA; 2 Department of Psychology, Georgia State University, P.O. Box 5010, Atlanta, GA 30302 USA

**Keywords:** Illusory truth, Repetition, Fluency, Belief, Truthfulness

## Abstract

Repeated information is often perceived as more truthful than new information. This finding is known as the illusory truth effect, and it is typically thought to occur because repetition increases processing fluency. Because fluency and truth are frequently correlated in the real world, people learn to use processing fluency as a marker for truthfulness. Although the illusory truth effect is a robust phenomenon, almost all studies examining it have used three or fewer repetitions. To address this limitation, we conducted two experiments using a larger number of repetitions. In Experiment 1, we showed participants trivia statements up to 9 times and in Experiment 2 statements were shown up to 27 times. Later, participants rated the truthfulness of the previously seen statements and of new statements. In both experiments, we found that perceived truthfulness increased as the number of repetitions increased. However, these truth rating increases were logarithmic in shape. The largest increase in perceived truth came from encountering a statement for the second time, and beyond this were incrementally smaller increases in perceived truth for each additional repetition. These findings add to our theoretical understanding of the illusory truth effect and have applications for advertising, politics, and the propagation of “fake news.”

## Significance statement

Repetition can affect beliefs about truth. People tend to perceive claims as truer if they have been exposed to them before. This is known as the illusory truth effect, and it helps explain why advertisements and propaganda work, and also why people believe fake news to be true. Although a large number of studies have shown that the illusory truth effect occurs, very little research has used more than three repetitions. However, in the real world, claims are often encountered at much higher repetition rates. The goal of the current research was to examine how a larger number of repeated exposures affects our judgments of truth. To do so, we conducted two experiments. In each experiment, we asked participants to read trivia statements such as “*The gestation period of a giraffe is 425 days”*. In Experiment 1, the trivia statements were shown either 1, 3, 5, 7, or 9 times. In Experiment 2, the trivia statements were shown either 1, 9, 18, or 27 times. One week later, we showed participants these same facts along with new facts and asked them to rate their truthfulness. In both experiments, we found that the more often that participants had previously encountered the trivia statement, the more truthful they rated it to be, but the largest increases in perceived truth occurred when people encountered a statement for the second time. Together these experiments show the powerful effect of simple repetition in affecting our judgments of truth.

## The illusory truth effect

Not everything that we believe is true. For example, according to a recent survey of teachers in Great Britain and The Netherlands, 48 percent and 46 percent, respectively, falsely believed that people only use ten percent of their brains (Dekker et al. 2012; see also van Dijk and Lane 2020). Problematically, as a result of this false belief, some people also have the misperception that “a little brain damage” is unimportant (Guilmette and Paglia 2004).

More recently, there has been concern about the consequences of peoples’ beliefs in misinformation, fake news, and conspiracy theories about the coronavirus disease (COVID-19) pandemic. In response to this health crisis, false information has been widely circulated. In fact, during the early stages of the outbreak, an analysis of posts to the social media platform Twitter showed that nearly a quarter of all COVID-19 tweets contained misinformation (Kouzy et al. 2020). As one concrete example, during the early days of outbreak, the Belgian newspaper *Het Laastste Nieuws* published an article suggesting that 5G, the cellular communication standard, might be linked to the development of COVID-19. Although this idea is not supported by science, this claim has since been repeated multiple times in other forums (Ahmed et al. 2020), and a survey in the spring of 2020, showed that 5 percent of UK residents believed that the symptoms of COVID-19 were linked to 5G mobile network radiation (Allington et al. 2020). Problematically, belief in this conspiracy theory was also associated with reduced health-protective behaviors (Allington et al. 2020), and since the initial newspaper article was published, there have been 77 reported attacks on cellular towers in the UK and over 40 attacks on cellular repair workers (Reichert 2020).

Why do beliefs in myths, misinformation and fake news persist, despite having been clearly disproven? One contributing factor is likely the fact that people have been exposed to this information repeatedly. Consistent with this idea, research has shown that repeated information is perceived as more truthful than new information. This finding is known as the illusory truth effect (for a review, see Brashier and Marsh 2020) and was first reported by Hasher et al. (1977). In this experiment, participants were exposed to a list of plausible statements, some of which were true (e.g., *Lithium is the lightest of all metals*) and some of which were false (e.g., *The capybara is the largest of the marsupials).* Participants were asked to judge the truthfulness of each statement. This process was then repeated during a second and third session. However, during these subsequent sessions, half of the statements had been previously encountered during the previous session(s), while the other half had not been encountered before. Results showed that with each successive session, participants rated the repeated statements as more truthful than they had in the previous session. Furthermore, these repetition-related increases in perceived truth did not vary based upon the objective truth of the statements.

The illusory truth effect, which is sometimes also referred to as the repetition truth effect, has now been replicated many times, and a meta-analysis showed that when comparing verbatim repetitions to novel information it is a medium effect size (*d* = 0.53; Dechêne et al. 2010). The illusory truth effect has also been demonstrated using a variety of different stimuli, including trivia statements (e.g., Bacon 1979), fake news headlines (Pennycook et al. 2018), product claims (Johar and Roggeveen 2007), opinion statements (Arkes et al. 1989), rumors (DiFonzo et al. 2016), and misinformation about observed events (Zaragoza and Mitchell 1996). The effect occurs regardless of whether the time between the repetitions is minutes (Arkes et al. 1989), weeks (Hasher et al. 1977), or even months apart (Brown and Nix 1996). Furthermore, the effect does not depend upon the source of the statements (Begg et al. 1992) and occurs even when participants are explicitly told that the source of the statements is unreliable (Henkel and Mattson 2011) or when the initial statement had a qualifier that cast doubt on the statement’s validity (Stanley et al. 2019). Further evidence of the robustness of this effect comes from studies showing that the illusory truth effect even occurs when the repeated statements are highly implausible (e.g., *The earth is a perfect square;* Fazio et al. 2019) or when the repeated statements directly contradict participants’ prior knowledge (e.g., *The fastest land animal is the leopard;* Fazio et al. 2015).

### Explanations of the illusory truth effect

A variety of different psychological explanations have been proposed to explain why repetition increases perceived truth (for a review, see Unkelbach et al. 2019). However, the most commonly cited explanation is the processing fluency account. Processing fluency refers to the metacognitive experience of ease or difficulty that accompanies a mental process (see Alter and Oppenheimer 2009). According to the processing fluency account, when information is repeated, it is processed more fluently and is consequently perceived to be more truthful (e.g., Unkelbach 2007; Unkelbach and Stahl 2009). This judgment occurs because we have learned over time that fluency (i.e., a proximal cue) is predictive of truthfulness (i.e., a more distal property that is not readily observable; Unkelbach and Greifeneder 2013). Support for the processing fluency account comes from other research showing that illusions of truth can occur even without repetition, such that people rate information presented in easy-to-read font (Reber and Schwarz 1999) or easy-to-understand speech (Lev-Ari and Keysar 2010) as being more truthful than information presented in a less perceptually fluent format.

A further explanation of why repetition increases processing fluency comes from Unkelbach and Rom’s (2017) referential theory of truth. In brief, this theory begins by noting that within a statement, the composite elements have preexisting degrees of semantic association with one another. Sometime references are already coherently linked with one another (e.g., “student” and “teacher”), but other times they are not (e.g., “sailor” and “secretary”). However, when a statement is repeated, this repetition serves to increase the coherence between the composite reference elements. This in turn results in the statement being processed more fluently and therefore perceived as more true. Thus, according to referential theory, processing fluency can be seen as an outcome of a memory network with coherent composite references (for further discussion, see Unkelbach et al. 2019).

When contemplating how repetition will affect memory coherence and/or processing fluency, it is also important to consider habituation effects. Habituation is a form of learning that occurs across species, and it refers to the fact that as the number of repetitions of a given stimulus increases there are exponential decreases in the frequency of the associated behavioral responses (for a review see Rankin et al. 2009). Habituation also occurs at the neural level in the form of repetition suppression effects. As the number of repetitions of a given stimulus increases, there are exponential decreases in the firing rates of the neurons (for a review see Grill-Spector et al. 2006). Repetition suppression effects are sometimes interpreted as an index of more fluent processing of semantic representations (e.g., Hasson et al. 2006; Henson 2003; Henson et al. 2002), which suggests that as the number of repetitions increases, the corresponding increases in processing fluency become incrementally smaller. This finding in turn has important implications for the perceived truth of these statements: As the number of repetitions of a statement increases, there should also be incrementally smaller increases in the perceived truth of that statement. The overarching goal of the current research was to test this hypothesis.

### Number of repetitions and perceived truth

Although a large body of research has shown that repeated information is perceived as more truthful than new information, to our knowledge only four prior studies have used more than three repetitions, and their conclusions have been mixed. Each of these prior studies is described in more detail below.

In a first study by Arkes et al. (1991, Experiment 3), participants were asked to judge the perceived truthfulness of statements across six different study sessions. As expected, results showed that perceived truthfulness was higher in the second session as compared to the first session. However, pairwise comparisons of the ratings given in the subsequent adjacent study sessions were not statistically significant. Based upon this, Arkes et al. (1991) concluded that further repetitions do not lead to further increases in perceived truthfulness.

However, other research has suggested that larger increases in the number of repetitions can still lead to increases in perceived truthfulness. In a study by Koch and Zerback (2013), participants were presented with the single statement “*microcredits reduced poverty in emerging nations*” either 1, 3, 5, or 7 times. These repetitions were embedded in a newspaper article describing an interview with the founder of the microcredit loan system. Structural equation modeling suggested that this statement was perceived as more truthful the more often that it was presented. However, this was obscured by the fact that in this context, repetition of this statement was also perceived to be a persuasion attempt, which in turn led to reactance and reduced belief in the statement’s truth.

Finally, two other studies suggest that there may be a logarithmic relationship between number of repetitions and perceived truth. First, Hawkins et al. (2001) observed increases in truth ratings up to 4 repetitions, but each increase was diminished from the last. Likewise, using a greater number of repetitions, DiFonzo et al. (2016) observed increases in truth ratings up to 6 repetitions (Experiments 1 and 2) and 9 repetitions (Experiment 3), with each repetition-related increase again being diminished from the last. However, conclusions from this study should be interpreted cautiously as only one statement was used per repetition condition, which may have reduced the reliability of the measure. Furthermore, these statements were presented as rumors within a narrative story, which could potentially have been perceived as a persuasion tactic, and hence reduced (rather than increased) perceived truth (Koch and Zerback 2013).

Thus, although we predict that increases in the number of repetitions should lead to logarithmic increases in perceived truthfulness, previous research examining this question has yielded contradictory conclusions, and the only two studies that have used more than 6 repetitions presented the information in a narrative context (DiFonzo et al. 2016; Koch and Zerback 2013). To further examine this question, we conducted two experiments that varied in the number of repetitions. In Experiment 1, the trivia statements were shown up to 9 times, whereas in Experiment 2, the trivia statements were shown up to 27 times. Within each experiment, we first tested for the presence of the illusory truth effect (i.e., are repeated statements perceived as more truthful than new statements?). We then tested our prediction that there is a logarithmic (as opposed to linear) relationship between repetition frequency and truth ratings.

## Experiment 1

### Power analysis and participants

An a priori power analysis using G*Power 3.1 that specified a matched-pair *t* test with an alpha level of 0.05, reported a minimum of 40 participants would be required to achieve 90% power to observe a medium-to-large effect of *d* = 0.53, which is the average effect size of the illusory truth effect reported in a prior meta-analysis (Dechêne et al. 2010). To account for attrition between the two study sessions (see Procedure section) and data exclusions, we aimed to have 100 participants complete Session 1. Participants were recruited using Amazon Mechanical Turk through the Turk Prime platform (www.cloudresearch.com; Litman et al. 2017). There were 153 individuals who consented to participate, but only 95 completed Session 1. One week later, 78 of these participants returned, but only 66 fully completed Session 2. Of these participants, we then excluded the 10 participants who failed one or more of the included attention checks (see Procedure section). This left a final sample size of 51 in the analyses reported below.

Participants were required to be residents of the USA and to be at least 18 years of age. The final sample (*M*_age_ = 33.27, *SD* = 7.81, range 20–55) consisted of 27 men and 24 women. They self-identified their race and ethnicity as follows: 39 identified as White or Caucasian, 9 as Black or African American, and 3 as Hispanic. Participants were also asked about their highest obtained level of education: 1 reported having a Ph.D., M.D., or J.D., 4 reported having a Master’s degree, 19 reported having a 4-year college Bachelor’s degree, 6 reported having a 2-year college degree, 14 reported having some college experience, and 7 reported having a high school diploma or equivalent.

### Materials and design

Stimuli consisted of a list of 100 trivia statements. Of these, 66 statements were adaptations of the questions from Nelson and Narens norms (1980) that were previously used by both Mutter et al. (1995) and by Henkel and Mattson (2011). Previous norming of this set of statements showed that they were relatively unknown, but that people perceived them as plausible (Mutter et al. 1995). Additional 34 trivia statements were found via online resources. These supplemental trivia statements were not normed, but were judged by the research team to also be plausible, but relatively unknown (e.g., *The zipper was invented in Norway*). Thus, the truthfulness of the statements used in the current research was ambiguous, which should increase the magnitude of the illusory truth effect (Fazio et al. 2019).

Whereas some prior studies have included both true and false statements, research has shown that repetition exerts equivalent increases in the perceived truth of previously unknown true and previously unknown false statements (e.g., Hasher et al. 1977; Pennycook et al. 2018, Experiment 2), and repetition even increases the perceived truth of false statements that directly contradict prior knowledge (Fazio and Sherry 2020; Fazio et al. 2015). Given that the truth value of our chosen statements was expected to be largely unknown to participants, and hence the veracity of the statements should not affect the repetition-related increases in perceived truth, we opted to only use factually accurate statements. In addition, because it can be difficult to reduce people’s belief that previously encountered misinformation is true (for a review see Lewandowsky et al. 2012), our use of only factually accurate statements also ensured that participants did develop false beliefs as a result of participation in this study.

For counterbalancing purposes, the 100 trivia statements were divided into 10 sets of 10 statements. In doing so, we ensured that statements pertaining to particular categories (e.g., geography facts) were distributed across the 10 sets. For each participant, 5 sets of facts were seen during Session 1 and corresponded to the five repetition conditions (one, three, five, seven, and nine). During the Session 2 truth ratings (see Procedure section), all 10 sets of facts were shown: Five sets of facts were new items that did not previously appear during Session 1 and the other five sets of facts were previously seen during Session 1. Counterbalancing was used such that across all participants, each set of facts appeared equally often as a repeated and new item, and when it was a repeated item, it appeared equally often across the five repetition conditions. This resulted in ten different counterbalanced versions of the experiment.^1^

### Procedure

All procedures were approved by the Institutional Review Board (IRB) at Georgia State University (protocol H19217). Participation in this experiment consisted of two separate sessions, separated by one week. Each session was completed online, using either a computer or a mobile device.

*Session 1* During Session 1, participants were randomly assigned to one of ten versions of the experiment, which represented the counterbalancing of specific trivia statements across repetition type and session (see Materials and Design section). Participants then provided consent and completed a demographics questionnaire. Participants next saw a series of trivia statements and rated how interesting they found each statement. They were instructed that some trivia statements would be shown more than once; however, for each statement they should rate how interesting they found it at that very moment. The participants then saw the trivia statements one at a time in a random order. Each trial consisted of the presentation of the trivia statement for 4 s. After this, the statement disappeared from view and participants were asked to rate how interesting the statement was on a scale of 1 (not interesting) to 6 (very interesting). These ratings were self-paced.

Over the course of this Session 1 task, participants saw 50 statements. Each statement was presented either one, three, five, seven, or nine time(s) and in total there were 10 statements in each repetition condition. This made for 250 trials. Additionally, three attention check trials were also included. These attention check trials simply stated: *Please select X for the next rating*, with X being either the answer choice of *1*, *2*, or *3*. The order of the 253 trials was randomized (although the precise randomization order that was used for each participant was not recorded). On average, participants spent 54.87 min completing the Session 1 tasks and were compensated $4.50 for their participation.

*Session 2* Consistent with previous research (e.g., Arkes 1989; Boehm 1994; Fazio et al. 2015), one week later participants were invited to complete a second study session. During Session 2, participants were shown our entire list of 100 statements. Of these, 50 had been previously seen during Session 1 and 50 were new statements. We chose to use a mixed-list of repeated and new facts as this should create variability in the fluency of the statements, which should increase the likelihood of observing illusory truth effects (e.g., Dechene et al. 2009; Garcia-Marques et al. 2019). The 100 statements were presented in a random order, one at a time, and participants were asked to rate how truthful they found each statement on a scale of 1 (not truthful) to 6 (very truthful). Participants were instructed that we were interested in their own perceptions about the truthfulness of the statements, and were told not to look up any of the statements while completing the task. During Session 2, two attention check trials, similar to those used in Session 1, were also included. On average, participants spent 13.78 min completing the Session 2 tasks and were compensated $3.50 for their participation.

### Results

We first evaluated whether or not we replicated the illusory truth effect. As in prior research, in a matched-pair *t* test, we found that repeated statements elicited higher truth ratings (*M* = 4.49, *SD* = 0.60; collapsing across repetition conditions) compared to the never-before-seen statements (*M* = 3.76, *SD* = 0.67), *t*(50) = 7.16, *p* < 0.001, *d* = 1.00.^2^

**Fig. 1:**
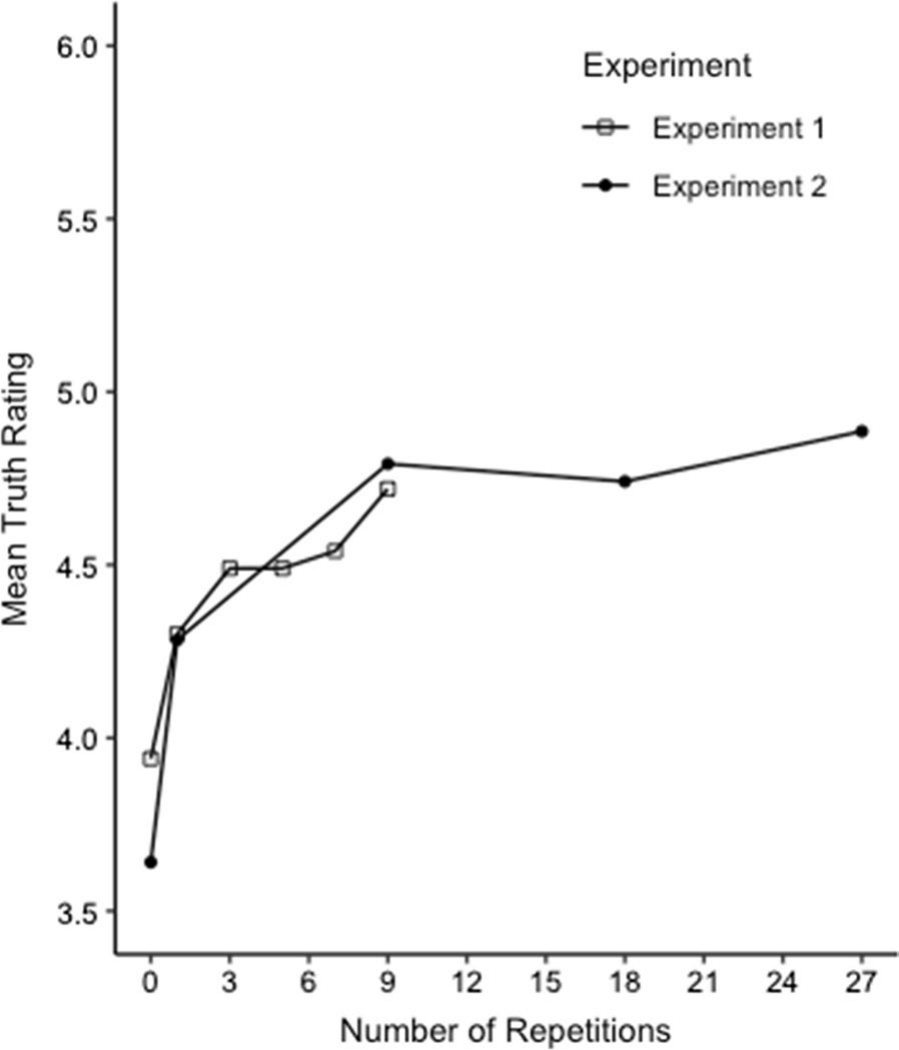
Mean Truth Ratings as a Function of Number of Repetitions in Experiment 1 and Experiment 2

We next evaluated our hypothesis that there would be a logarithmic (as opposed to linear) relationship between the number of times a statement was repeated during Session 1 and perceptions of truth during Session 2. To do so, for each participant we calculated the correlation coefficient between their Session 2 truth ratings and the number of Session 1 repetitions (0, 1, 3, 5, 7, 9), and also between their Session 2 truth ratings and the log of the number of Session 1 repetitions (for a similar procedure, see Guild et al. 2014). In both cases, we added a constant of 1 to the number of Session 1 repetitions, to account for the fact that the log of 0 is undefined.^3^ On average, truth ratings had a moderate-to-large correlation with the linear scaling of the Session 1 repetitions (mean *r* = 0.46, *SD* = 0.43; range of *r*s = -0.72 to 0.93; correlations were greater than zero for 88% of participants). Truth ratings also had a large correlation with the logarithmic scaling of the Session 1 repetitions (mean *r* = 0.52, *SD* = 0.44, range of *r*s = -0.57 to 0.98; correlations were greater than zero for 84% of participants). However, a matched pair *t*-test showed that the magnitude of the correlation was significantly greater when using the logarithmic scale, as compared to the linear scale, *t*(50) = 4.83, *p* < 0.001, *d* = 0.67 (see Fig. 1).

As shown in Table 1, follow-up Bonferroni-adjusted pairwise comparisons showed that there were large differences in perceived truth between the never-before-seen items and the previously seen items. The new statements were rated as significantly less truthful than statements in the 1, 3, 5, 7, and 9 repetition conditions (i.e., there was an illusory truth effect). However, there were fewer significant differences between items from the other repetition conditions. In fact, the only other significant pairwise comparison was between statements in the 1 and 9 repetition conditions.

**Table 1:** Experiment 1 truth ratings as a function of repetition condition and the pairwise comparison effect sizes between repetition conditions

	Number of Session 1 repetitions					
	0 (new)	1	3	5	7	9
*M*s and *SD*s of truth ratings	*M* = 3.76 *SD* = 0.67	*M* = 4.27 *SD* = 0.71	*M* = 4.51 *SD* = 0.72	*M* = 4.49 *SD* = 0.71	*M* = 4.49 *SD* = 0.81	*M* = 4.69 *SD* = 0.72
0 (new)	–	*d* = 0.59*	*d* = 0.91*	*d* = 1.02*	*d* = 0.81*	*d* = 1.09*
1		–	*d* = 0.29	*d* = 0.29	*d* = 0.26	*d* = .58*
3			–	*d* = 0.04	*d* = 0.02	*d* = 0.29
5				–	*d* = 0.01	*d* = 0.38
7					–	*d* = 0.37
9						–

*Note*: Bonferroni-adjusted post hoc comparisons were used to compare the repetition conditions. Significant differences between conditions (*p* < .05) are indicated in bold (and also with an asterisk)

## Experiment 2

Although Experiment 1 showed that increased repetitions were associated with logarithmic increases in truth ratings, one limitation of this study is that our maximum number of repetitions was nine. To address this, in Experiment 2 we repeated the facts either 1, 9, 18, or 27 times. We chose intervals of 9 repetitions because the Experiment 1 results showed a significant difference in the perceived truthfulness of items previously presented once versus nine times.

In Experiment 2, we also limited our sample to younger adults, aged 18 to 35. Prior research has shown that older adults demonstrate greater illusory truth effects than younger adults (Law et al. 1998). Replicating this, in Experiment 1 we also found that the difference in truth ratings between old and new items was larger with increasing age, *F*(1, 49) = 7.05, *p* = 0.011, *η*_*p*_^*2*^ = 0.13. Although none of the Experiment 1 conclusions change when including age as a factor (i.e., regardless of whether participants were relatively older or relatively younger, in Experiment 1 the logarithmic scale was more strongly related to truth ratings than the linear scale), to reduce variability in illusory truth effects, in Experiment 2 we limited our sample to individuals aged 18 to 35.

### Participants

Using the same recruitment strategies as in Experiment 1, there were 151 individuals consented to participate in Experiment 2, but only 100 completed Session 1. One week later, 70 of these participants returned, but only 64 fully completed Session 2. As in Experiment 1, we then excluded the 7 participants who failed one or more of the included attention checks (see Procedure section). This left a final sample size of 57 in the analyses reported below.

Participants were required to be residents of the USA, aged 18 to 35. Within the final sample, participants were on average 29.46 years old (*SD* = 3.49, range 21–35). Although all participants reported their age, due to experimenter error we did not assess gender, racial identity, or educational attainment in all eight of the counterbalanced versions of the experiment (see Materials and Design section). Gender was only assessed in two versions: Of participants asked this question there were 8 men and 8 women. Race was assessed in seven versions, with these participants self-identifying as follows: 38 as White or Caucasian, 5 as Black or African American, 3 as Asian, 1 as American Indian or Alaska Native, 1 as Biracial, and 2 did not identify with any of the provided racial identity choices. Educational attainment was only assessed in two versions: Of participants asked this question 6 reported having a 4-year college Bachelor’s degree, 2 reported having a 2-year college degree, 3 reported having some college experience, 5 reported having a high school diploma or equivalent, and 1 reported having some high school.

### Materials and design

The list of 100 statements used in Experiment 1 was pared down to 64 statements. This was done pseudorandomly with the constraint that we maintained diversity in the broad categories of trivia facts represented. For instance, we ensured that we were not discarding all of the statements related to animals or all of the statements related to geography. The statements were then divided into eight sets of eight statements. For each participant, four sets were used during Session 1 and corresponded to the four repetition types (1, 9, 18, and 27). The other four sets were used as new items during Session 2. Counterbalancing was used such that across participants, each set appeared equally often as a repeated or new items, and when it appeared as a repeated item, appeared equally often across the four repetition types. This resulted in eight different counterbalanced versions of the experiment.

### Procedure

The procedure for Experiment 2 was approved by the IRB at Georgia State University (protocol H19217) and was identical to Experiment 1 with the following exceptions. First, the ratings during Session 1 were not self-paced. In order to standardize the amount of time spent viewing the statements, participants were given 4 s to view each fact, followed by 4 s to rate their current interest in the fact.

Second, as noted above (see Materials and Design), during Session 1 of Experiment 2 the statements were presented either 1, 9, 18, or 27 time(s). As there were 8 statements in each repetition condition, this made for 440 critical trials. With the addition of 3 attention trials, the total number of trials was 443 (as opposed to 253 in Experiment 1).

Third, at the end of Session 2 we also asked participants whether they had looked up, or discussed with others, any of the Session 1 facts during the prior week. Only three participants reported having done so, and these participants further reported that this affected two or fewer of the Session 1 facts. Excluding these participants did not change any of the reported patterns of results, and hence, they were retained in the subsequent analyses.

On average, participants spent 68 min completing Session 1 and 13.84 min completing Session 2 and compensated $7.25 and $3.75, respectively.^4^

### Results

We first tested for the illusory truth effect using a matched-pairs *t* test. Results showed that the repeated statements (*M* = 4.66, *SD* = 0.86; collapsing across repetition conditions) elicited higher truth ratings compared to never-before-seen statements (*M* = 3.64, *SD* = 0.65), *t*(56) = 8.22, *p* < 0.001, *d* = 1.09.

We next tested our hypothesis that there would be a logarithmic (as opposed to linear) relationship between the number of times a statement was repeated during Session 1 and perceptions of truth during Session 2. As in Experiment 1, we correlated each participants’ average truth ratings during Session 2 with both the number of Session 1 repetitions (0, 1, 9, 18, 27), as well as with the log of the number of Session 1 repetitions. In both cases, we added a constant of 1 to the number of Session 1 repetitions, to account for the fact that the log of 0 is undefined. As in Experiment 1, truth ratings tended to have a moderate-to-large correlation with the linear scaling of the Session 1 repetitions (mean *r* = 0.47, *SD* = 0.43; range of *r*s = -0.84 to 0.95; correlations were greater than zero for 82% of participants). Truth ratings also tended to have a moderate-to-large correlation with the logarithmic scaling of the Session 1 repetitions (mean *r* = 0.56, *SD* = 0.44, range of *r*s = -0.82 to 0.99; correlations were greater than zero for 86% of participants). However, a matched-pair *t* test showed that the showed that magnitude of the correlation was significantly greater when using the logarithmic scale, as compared to the linear scale, *t*(56) = 8.22, *p* < 0.001, *d* = 0.63 (see Fig. 1)

As shown in Table 2, this conclusion was further supported by follow-up Bonferroni-adjusted pairwise comparisons. Here, we found that new statements were rated as less truthful than those previously seen 1, 9, 18, or 27 times. However, there were very few statistically significant differences between items from the other repetition conditions. Statements in the 1 repetition condition were rated significantly less truthful than statements in the 9, 18, and 27 repetition conditions. However, no other comparisons between repetition conditions were found to be statistically significant.

**Table 2:** Experiment 2 truth ratings as a function of repetition condition and the pairwise comparison effect sizes between repetition conditions

	Number of Session 1 repetitions				
	0 (new)	1	9	18	27
*M*s and *SD*s of truth ratings	*M* = 3.64 *SD* = 0.65	*M* = 4.26 *SD* = 0.83	*M* = 4.78 *SD* = 1.01	*M* = 4.72 *SD* = 1.02	*M* = 4.87 *SD* = 0.99
0 (new)	–	*d* = 0.88*	*d* = 1.02*	*d* = 0.96*	*d* = 1.13*
1		–	*d* = 0.62*	*d* = 0.49*	*d* = 0.80*
9			–	*d* = 0.11	*d* = 0.17
18				–	*d* = 0.28
27					–

*Note: *Average truth ratings, and their standard deviations, are presented in the first row on this table. Bonferroni-adjusted post hoc comparisons were used to compare the repetition conditions. Significant differences between conditions (*p* < .05) are indicated in bold (and also with an asterisk)

## Discussion

The goal of this research was to test the hypothesis that the more frequently information is encountered, the more truthful that information is perceived to be, and that this relationship is logarithmic in nature. To test this, we asked participants to read trivia statements, which were repeated up to 9 times in Experiment 1 and up to 27 times in Experiment 2. One week later, participants saw these same trivia statements alongside the new statements and were asked to judge the truthfulness of each statement. As expected, in both experiments we replicated the illusory truth effect such that repeated statements were perceived as more truthful than new statements. We also found that perceived truthfulness increased as the number of repetitions increased, and in line with our predictions, these increases were logarithmic in nature. In both experiments, the largest increases in perceived truth came from encountering a statement for the second time. However, beyond this, there were progressively smaller increases in perceived truth for each additional repetition, which were not statistically significant beyond 9 repetitions.

These findings support the predictions based upon both the processing fluency account and also based upon the referential theory of truth. They are also consistent with research by Hawkins et al. (2001) who found that repeating information up to 4 times results in progressively smaller increases in truth ratings. Likewise, DiFonzo et al. (2016) found a logarithmic relationship, such that repeating information up to 9 times also results in progressively smaller increases in truth ratings. We replicate their findings using a larger number of items outside of a narrative context (Experiment 1) and extend their results by showing that this pattern continues up to at least 27 repetitions (Experiment 2).

In addition, our results—but not our conclusions—are also consistent with those reported by Arkes et al. (1991, Experiment 3). As in their study, we found that even though information shown for the second time was rated as significantly more truthful than new information, pairwise comparisons of truth ratings for the subsequent repetition conditions were rarely statistically significant. For instance, in our Experiment 1, the truth ratings for the statements presented 3 times did not significantly differ from the truth ratings for the statements presented 5 times (see Tables 1, 2). Based upon similar null results, Arkes et al. (1991) concluded that while a first repetition increases perceived truth, subsequent repetitions do not. In contrast, we conclude that while a first repetition produces the largest increase in perceived truth, subsequent repetitions produce subsequent increases in truth that are incrementally diminished in size. As a result, statistically significant increases in perceived truth may only occur after a large number of additional repetitions. Furthermore, because a logarithmic function has no asymptote, theoretically, it stands to reason that repetitions will elicit higher and higher truth ratings indefinitely. However, at some point these incremental increases in perceived truth will become so small in magnitude that they no longer have practical value.

Understanding the practical value of increased repetitions is important because the illusory truth effect affects important daily life decisions (for further discussion, see Unkelbach et al. 2019) and our findings are highly relevant within the realms of politics and “fake news.” For example, using actual fake-news headlines from the 2016 US presidential election, Pennycook et al. (2018) found that the more often that participants were exposed to these headlines, the more likely they were to believe them to be true. This occurred even when the headlines were clearly tagged as being false facts, and when their content was inconsistent with the participants’ own political ideology. Although this demonstrates that a single encounter with a fake news story will make it seem more truthful, in our daily lives we sometimes encounter false information repeatedly. For example, during his 2016 campaign to be elected as President of the USA, Donald Trump stated 86 times that the construction of a wall between the USA and Mexico had already begun (see Murray et al. 2020). Although this was false, our results suggest that each time this claim was repeated, its perceived truthfulness incrementally increased.

These results are also relevant for understanding the public’s response to the COVID-19 pandemic: Our results suggest that the more often messages about COVID-19 are repeated, the more truthful they will be perceived. The consequences of this can be positive or negative, depending upon the validity of the messages. An example of this comes from Bursztyn, Rao, Roth, and Yanagizawa-Drott’s (2020) analyses of the relationship between viewers’ health outcomes and the coverage of COVID-19 they had seen on *Hannity* and *Tucker Carlson Tonight.* Although these cable news shows are both broadcast on Fox News, beginning in early February of 2020, Carlson warned viewers that COVID-19 might pose a serious health threat to the USA. In contrast, Hannity originally claimed that COVID-19 was no different than the flu and was being used by Democrats as a political weapon. Hannity only began to describe COVID-19 as a threat in mid-March of 2020. Being exposed to these repeated messages was associated with adverse health outcomes for the Hannity viewers. In a survey of Fox News viewers aged 55 of older in April 2020, a one standard deviation higher viewership of Hannity (relative to Carlson) was associated with 33% more COVID-19 cases on March 14th, and 34% more COVID-19 deaths on April 4th. Presumably this occurred because the messages about COVID-19 had been repeatedly presented on the news, and were believed by the viewers. This in turn may have had a ripple effect, as people are also more likely to share with others information that they have repeatedly encountered (Effron and Raj 2020).

A final domain for which the current experiments’ findings are relevant is advertising. Prior research has shown that repeated advertisements are associated with people perceiving the advertised product as higher in quality (Moorthy and Hawkins 2005), and our results suggest that it may also increase perceived truth of the advertisement message. However, one factor that often moderates advertising repetition effects is the number of advertisements (e.g., Burton et al. 2019; Kohli et al. 2005). For instance, results of a meta-analysis suggest that there are increases in positive attitudes with up to 10 exposures of an advertisement, after which there are decreases in positive attitudes (Schmidt and Eisend 2015). The terms “wear-in” and “wear-out” are used to describe these effects. An advertisement is “worn in” when the repetition initially garners a positive effect and is “worn out” when the repetition produces no effect or even a negative one (Pechmann and Stewart 1988).

Consistent with this idea, data from Experiment 2 suggest that repetition-related increases in perceived truth may be “worn out” after 9 repetitions. As shown in Fig. 1, after 9 repetitions the truth ratings appear to approach an asymptote, and after this point the practical value of further repetitions may be limited. Although we did not observe any evidence that repetitions beyond this negatively affect perceived truth, it is possible that an inverse U-shape may have occurred if we had used a persuasion context (such as would occur during advertising). This is consistent with prior research from Koch and Zerback (2013). As previously described, participants in this study read a newspaper interview with the founder of microcredit loans. Embedded in this interview was the statement “*microcredits reduced poverty in emerging nations,”* which was repeated either one, three, five, or seven times. Results from a structural equation model suggested that increased repetitions lead to increased belief that microcredit loans decrease poverty in emerging nations. However, increased repetitions also led participants to trust the communicator less, and to believe that the message was a persuasion attempt. As a result, participants who heard statements multiple times interpreted the reason for those repetitions as an intent to persuade them, and demonstrated reactance by rating the statement lower in truthfulness.

It is also possible that we did not observe an inverse U-shaped curve because we did not include a sufficient number of repetitions. Support for this possibility comes from research on the mere-exposure effect. This is the finding that repeated exposure to an initially neutral and unfamiliar stimulus results in greater liking of that stimulus (Zajonc 1968), and this is thought to reflect repetition-related increases processing fluency (Reber and Schwarz 2001; Reber et al. 1998). However, a meta-analysis shows that the relationship between repetition and liking resembles an inverted U-shaped curve. More specifically, liking continues to increase up to about 62 repetitions, but after this point additional repetitions lead to declines in liking (Montoya et al. 2017; see also Bornstein and D’Agostino 1992). If a peak in perceived truth occurs after a similar number of repetitions, the current experiments would not have observed it. Statements were repeated a maximum of 9 times in Experiment 1 and 27 times in Experiment 2. Thus, future research examining the relationship between repetition and perceived truth should include an even greater number of repetitions.

Future studies should also address the limitations that were present in these experiments. The first being that we did not assess whether or not any of the statements included were previously known to each participant. While we could have assessed pre-experimental knowledge of the facts, it has been shown that prior knowledge does not shield one from the illusory truth effect (Fazio et al. 2015). It is therefore likely that the patterns reported here would have emerged even for misinformation or fake news that contradicted prior knowledge.

A second limitation has to do with the presentation and length of the study sessions. In these studies, participants read trivia statements in black text on a white background for over an hour on their phones or computers. This may have contributed to mind-wandering and boredom, and even though all participants included in analyses passed our attention checks, they may not have given the statements their full attention. This reduced attentiveness may actually have maximized the illusory truth effects that were observed. For instance, Hawkins and Hoch (1992) found what they termed “low-involvement” learning was a key factor to observing the illusory truth effect. When participants were exposed to advertising statements, those who engaged in the “low-involvement” learning task (i.e., those who were asked to rate the statements based on how easy they were to understand) experienced stronger subsequent illusory truth effects than those in the “high-involvement” learning task (i.e., those who were asked to rate statements based on how truthful they were). It appears that deeper engagement while processing the statement can protect one from repetition-based illusory truth effects. Consistent with this, Brashier et al. (2020) recently found that participants who were actively involved in “fact-checking” the presented statements showed a reduced illusory truth effect (at least when they had the requisite knowledge to perform the task).

A final limitation is that we did not examine the role of repetition spacing in modulating the magnitude of the illusory truth effect. In the current experiments, the trivia facts (and their repetitions) were presented in a random order for each participant during the first study session, but unfortunately these randomization orders were not recorded. Given prior research showing that neural repetition suppression is reduced for spaced, as compared to massed, repetitions (e.g., Xue et al. 2011), it is reasonable to hypothesize that illusory truth effects should also be greater following spaced, as compared to massed, repetitions. Preliminary results from our laboratory support this hypothesis (Barber et al. 2020), and ongoing research is now examining the combined influence of the number of repetitions and the spacing of those repetitions in affecting perceived truth.

In summary, our results suggest that the more often information is repeated, the more likely it is to be believed. This is important since we often encounter information whose validity is unknown. Although believing repeated information to be true is evolutionarily efficient in a context where most of the information encountered is correct, it can be detrimental to believe information that is incorrect. Sometimes these consequences can be trite: If you are repeatedly shown the false statement “*Salty water boils faster,”* you may come to believe this to be true. However, acting on this false belief will only slightly elongate your cooking time. In contrast, other times the consequences can be life-threatening: If you are repeatedly told that *“COVID-19 is no more dangerous than the common cold,”* you may come to believe this to be true, but acting on this false belief may increase your risk of infection and death. Although our studies did not use fake news, conspiracy theories, or misinformation for stimuli, our results shed light on the mechanism underlying illusory truth effects, and suggest that repeated exposures likely lead to increased belief. In addition, our results suggest that the largest increases in perceived truth come from hearing information a second time. Going beyond this, subsequent repetitions lead to progressively smaller increases in perceived truth. However, after 9 repetitions these increases may no longer be practically meaningful.

## Data Availability

Data and study materials are available from the corresponding author upon request.

## Footnotes

1 Due to attrition and data exclusions, the counterbalanced versions of the statements were not evenly represented in the final samples of Experiment 1 or Experiment 2. However, when including counterbalance version number as a factor in analyses, there were no main effects or interactions to report. In addition, the sets of facts did not significantly differ in the truth ratings they received the first time they were shown. Counterbalance version will therefore not be discussed further.

2 Within-subject effect sizes were determined using an online calculator available at https://memory.psych.mun.ca/models/stats/effect_size.shtml, which uses the formula *d* =|*m*1 − *m*2| / [*s*21 + *s*22 − (2*rs*1*s*2)].

3 As a concrete example, assume that a participant’s average truth rating for the new items (seen 0 times during Session 1) was 3.75. Also assume that this same participant’s average truth ratings for the repeated items were 4.6, 5.9, 6.0, 5.7, and 6.0 for items seen 1, 3, 5, 7, and 9 times during Session 1, respectively. Adding a constant of 1 to each repetition condition, this participant’s truth ratings would have a correlation of *r* = .80 with the linear number of Session 1 repetitions (1, 2, 4, 6, 8, 10), but a correlation of *r* = .93 with the log of the number of Session 1 repetitions (1, 0.30, 0.60, 0.78, 0.90, 1.0).

4 In Experiment 1, participants took longer than expected to complete Session 1. In Experiment 2 we standardized the amount of time spent rating the Session 1 statements, and increased the compensation to better reflect the amount of time that participants spent completing the task.
